# Empowering Better End of Life Dementia Care (EMBED‐Care): Co‐design of a digital holistic assessment and clinical decision support framework

**DOI:** 10.1002/alz.088707

**Published:** 2025-01-09

**Authors:** Nathan Davies, Clare Ellis‐Smith, Juliet Gillam, Tofunmi Aworinde, Charlotte Kenten, Catherine Evans, Elizabeth L Sampson

**Affiliations:** ^1^ University College London, London, London United Kingdom; ^2^ King’s College London, London, London United Kingdom; ^3^ University College London, Tottenham Court Road, London, London United Kingdom; ^4^ Royal London Hospital, East London NHS Foundation Trust, London United Kingdom

## Abstract

**Background:**

People with dementia have complex palliative care needs including psycho‐social, physical and spiritual; however, they are often unmet. It is important to empower people with dementia, family caregivers and professionals to work together to better assess and monitor ongoing needs. This study aimed to co‐design and test the feasibility of an integrated model of palliative dementia care to support holistic assessment and decision making for care in the community and care homes (assisted living facilities).

**Method:**

Using a systematic and iterative approach we co‐designed the EMBED‐Care framework, a holistic assessment and clinical decision support system hosted on a digital application. Using framework analysis we synthesised evidence from systematic reviews, large data, and a cohort study to identify unmet palliative care needs and concerns of people living with dementia. We presented the data in workshops, using nominal group techniques with people with dementia, family caregivers, and care professionals, to identify targets, components, and design of the framework. We conducted user testing with all groups to refine the framework. We co‐designed training and support to implement the framework, including bitesize animated videos, manuals, in‐person training sessions, local champions, and a telephone support system. The framework is currently being tested in a feasibility trial in two care homes and two community nursing teams.

**Result:**

The framework consists of five components: holistic assessment using the Integrated Palliative Outcome Scale for People with Dementia (IPOS‐Dem); alert system linked to IPOS‐Dem scores, priority setting; clinical decision support tools; and training programme. The IPOS‐Dem helps identify areas of concern and unmet need, providing opportunities for shared decision making and identify priorities of care. Each concern is linked to a decision support tool to support decision making and to address priorities. Alerts and reminders communicate concerns among teams and carers. A desktop version (dashboard) is available for professionals to view all their residents/patients, including IPOS‐Dem results, allowing symptom tracking. Findings from the feasibility study will be presented.

**Conclusion:**

This is the first intervention to link IPOS‐Dem with practical decision support to help break down the complexity of palliative dementia care, providing the right care at the right time.

